# Cardiovascular Risks and Benefits of Medications Used for Weight Loss

**DOI:** 10.3389/fendo.2019.00883

**Published:** 2020-01-15

**Authors:** Carolyn T. Bramante, Sarah Raatz, Eric M. Bomberg, Megan M. Oberle, Justin R. Ryder

**Affiliations:** ^1^Center for Pediatric Obesity Medicine, University of Minnesota Medical School, Minneapolis, MN, United States; ^2^Division of General Internal Medicine, University of Minnesota Medical School, Minneapolis, MN, United States; ^3^Division of Pediatric Endocrinology, University of Minnesota Medical School, Minneapolis, MN, United States; ^4^Division of Pediatric Epidemiology and Clinical Research, University of Minnesota Medical School, Minneapolis, MN, United States

**Keywords:** obesity, pediatric obesity, anti-obesity agents, cardiovascular diseases, blood pressure, diabetes mellitus type 2, obesity therapy

## Abstract

Obesity is a complex disease influenced by many neurohormonal pathways which regulate body weight toward homeostasis. Presently, the disease of obesity effects over a billion individuals worldwide with scalable treatment options in dire need. Pharmacologic interventions for obesity have been developed to help promote weight loss in individuals with obesity. This area is rapidly developing and will only exponentially increase to serve the demand for persons with obesity seeking biologically orientated solutions to treat their disease. Therefore, understanding the cardiovascular risks and benefits of these weight loss medications is of particularly importance due to obesities strong association with cardiovascular (CV) disease risk. Moreover, past experiences with pharmacotherapy agents with weight loss properties have demonstrated an association with adverse CV outcomes, leading to market removal, in most cases and concerns over using similar medications. To better understand the CV risks and benefits pharmacotherapy agents used for weight loss, this review will discuss medications which are FDA-approved for weight loss, as well as medications commonly used off-label for this indication. The goal is to provide an overview of the risks and benefits many of these medications can offer to help guide clinical decision making and patient education.

## Introduction

It is estimated that obesity afflicts 650 million adults and 380 million children worldwide (1). The leading cause of death and contributor to disability-adjusted life-years among adults with obesity remains cardiovascular (CV) disease (2). Due to the high prevalence of CV disease in persons with excess adiposity, it is important to understand the potential CV risks and benefits of anti-obesity medications. The need for these studies is further underscored by historical examples in which some anti-obesity medications were associated with significant CV complications. One such example is the combined use of fenfluramine and phentermine, which was associated with an increased development of valvular heart disease and pulmonary hypertension (3).

There are currently five medications, from a wide range of classes, that are Food and Drug Administration (FDA)-approved for long-term use (≥12 weeks) for obesity treatment: liraglutide (Saxenda), phentermine/topiramate (Qsymia), lorcaserin (Belviq), naltrexone/bupropion (Contrave), and orlistat (Xenical). Phentermine and diethylpropion are approved for short-term use (e.g., 12-weeks at a time). This review will outline the CV risks and benefits of anti-obesity medications, including those that are FDA approved for weight management and those that are commonly used off-label for their weight loss effects. We will largely focus on the CV risks and benefits of anti-obesity medications instead of other aspects of these medications, such as expected weight loss. Recent reviews of weight loss outcomes from these the anti-obesity medications have been published (4, 5).

## Diabetes Medications

Owing to the increased risk of CV complications among individuals with type 2 diabetes (T2D), in 2008 the FDA issued recommendations for randomized control trials to evaluate the CV risks of new medications for T2D (6). Here, we will cover the CV risks and benefits of multiple classes of diabetes medications, including data from the available FDA-required CV outcome trials (CVOTs). Medications covered include glucose-like peptide-1 (GLP-1) receptor agonists, of which liraglutide is FDA approved for the treatment of obesity in the absence of diabetes, as well as other classes of diabetes medications that have weight loss effects including dipeptidyl peptidase-4 (DPP-4) inhibitors, metformin, and sodium-glucose cotransporter 2 (SGLT2) inhibitors.

### Glucagon-Like Peptide-1 (GLP-1) Receptor Agonists

GLP-1 receptor agonists regulate glycemic control by stimulating insulin secretion in response to ingested nutrients, suppressing glucagon secretion, and delaying gastric emptying, which increases satiety and promotes weight loss (7). Liraglutide specifically has been shown to decrease parietal cortex activation for desirable food (8). Although liraglutide is the only GLP-1 receptor agonist that is currently FDA approved as an anti-obesity medication, this review will cover the CV risks and benefits of multiple GLP-1 receptor agonists, including liraglutide, semaglutide, exenatide, dulaglutide, and lixisenatide.

Liraglutide is approved for T2D management at a dose up to 1.8 mg per day (Victoza), and for weight loss at a dose of 3.0 mg/day (Saxenda). Liraglutide 3.0 mg daily for weight loss was assessed by a 56-week randomized placebo-controlled study [SCALE; (9)]. In this study, liraglutide was associated with the following changes compared to placebo: decreased SBP by 2.7 mmHg; decreased DPB by 0.7 mmHg; increased HR by 2.4 BPM; increased HDL by 1.7; decreased LDL by 2.0; and decreased triglycerides by 8.0 (9). This study was not a CVOT because it was not assessing the medication for use in diabetes, but the following outcomes occurred during the study: two non-fatal myocardial infarctions (MI) and one death from CV causes in the liraglutide group, and one non-fatal MI, one non-fatal stroke, and one death from CV causes in the placebo group. *Post-hoc* analysis of three randomized, double-blinded, placebo-controlled studies of liraglutide 3.0 mg daily showed a hazard ratio of 0.42 (95% confidence interval 0.17–1.08) for CV death, non-fatal MI, or non-fatal stroke, comparing liraglutide to comparator (10).

The LEADER trial (Liraglutide Effect and Action in Diabetes: Evaluation of Cardiovascular Outcome Results), was the FDA-mandated CVOT of liraglutide 1.8 mg daily for diabetes treatment in high-risk cardiovascular patients (11). The primary composite outcome of time to CV death, non-fatal MI, and non-fatal stroke occurred significantly less in the GLP-1 receptor agonist group compared to placebo (13 vs. 14.9%, *p* = 0.01 for superiority). The most significant decrease was noted in the incidence of CV death. Further, patients treated with liraglutide had fewer hospitalizations for heart failure, though the difference from placebo was not statistically significant. Similarly, both semaglutide and dulaglutide demonstrated lower risks for the same primary composite outcomes in their CVOT trials (12, 13). In comparison, the ELIXA trial for lixisenatide and the EXSCEL trial of weekly exenatide found these medications to be similar to placebo with regards to serious cardiovascular outcomes (14, 15).

These large CVOTs also examined for changes in key potential CV risk factors including heart rate (HR) and blood pressure (BP). In the LEADER trial, study participants in the liraglutide group had a significant mean increase in HR of 3 beats per minute (bpm) (95% CI, 2.5–3.4), decrease in systolic BP of 1.2 mmHg (95% CI, 1.9–0.5), and increase in diastolic BP of 0.6 mmHg (95% CI, 0.2–1.0). The trend of increased HR and decreased systolic BP supported findings seen in a prior meta-analysis (16). In patients with pre-existing, stable coronary artery disease, a 2017 study found that liraglutide led to increased heart rate (8 beats per minute), and decreased heart rate variability, thought to be due to effect on the sympathomimetic balance (17). A subsequent meta-analysis following the LEADER trial showed that the decrease in systolic BP observed with liraglutide did not maintain statistical significance after 1 year of treatment (18). Similar to liraglutide, the use of semaglutide and weekly exenatide, dulaglutide, and lixisenatide were also associated with a statistically significant but small increase in HR (1–2 bpm) and decrease in systolic BP in their respective large scale CVOTs (6–9). In patients recently hospitalized for heart failure with reduced ejection fraction, liraglutide did not improve nor worsen cardiovascular outcomes (19). Two recent meta-analyses showed that liraglutide improved lipid profiles with decreases in total cholesterol (TC), low-density lipoprotein (LDL), triglycerides (TG), and free fatty acids among patients with T2D (20, 21). Smaller studies, specifically examining liraglutide have also demonstrated CV benefits with reductions in LDL, waist circumference, and BP (22, 23). Overall, the safety profile of GLP-1 receptor agonists is favorable, and while they are associated with increases in HR, they have shown CV benefit with improvements in BP, lipids, CV death, non-fatal myocardial infarction, and non-fatal stroke.

### Sodium Glucose Co-transporter-2 (SGLT2) Inhibitors

The kidney processes about 180 liters of plasma each day, filtering many proteins including sodium and glucose. The proximal tubule of the kidney regulates the reabsorption of sodium and glucose via sodium glucose co-transporter-1 (SGLT1) and sodium glucose co-transporter-2 (SGLT2) which comprise 10–20% and 80–90% of reabsorption, respectively. By selectively inhibiting SGLT2, a downstream effect of glucose and sodium appearance in the urine is acutely observed. This effect is sustained long-term for glucose, but not sodium as salt and water balance becomes regulated.

Given these effects, SGLT2 inhibitors have demonstrated a wide-range of health benefits including CV risk reduction, vascular health improvement (reduction in BP and reduced arterial stiffness), weight loss, glycemic control, and improved insulin sensitivity (24–30). The SGLT2 inhibitor empagliflozin has demonstrated superiority in reducing the primary composite outcome of CV death, non-fatal MI and stroke compared to placebo in adults with T2D (31). While the mechanism(s) specific to CVD risk reduction remains unclear, glycosuria, the main mechanism of action, has been reported among non-diabetic adults under fasting and post-prandial conditions. Importantly, while the rates of glycosuria are lower in normal glucose tolerant adults compared to those with T2D, the relative amounts under both fasting and post-prandial conditions are still high enough to lead to an overall negative glucose and caloric deficit (32, 33) which provides the potential for downstream impact on CV risk factors in adults with obesity without the presence of T2D. Accompanying these metabolic changes (lowering of glucose and insulin with increases in glucagon), improvements in BP and arterial stiffness have been observed (34, 35). Moreover, the safety profile of SGLT2 inhibitors is acceptable with no CV side-effects observed.

### Metformin

The complete mechanism of action of metformin is not fully understood. However, known actions include its impact on decreasing hepatic gluconeogenesis, enhancing hepatic insulin sensitivity, increasing gut utilization of glucose, and potentially promoting GLP-1 secretion and altering the gut microbiome (36). With regard to CV risks or benefits of metformin, the UKPDS trial (United Kingdom Prospective Diabetes Study) favored metformin compared to diet therapy alone, showing that those receiving metformin had lower risk for macrovascular disease, including MI, sudden death, stroke, angina, and peripheral vascular disease (37). Other meta-analyses have not concluded there are statistically significant CV benefits of metformin on all-cause mortality, CV mortality, MI, peripheral vascular disease, or stroke (38, 39). Metformin has been shown to decrease TC (−11 ± 3 mg/dL), LDL (−11 ± 3 mg/dL), and triglycerides (−17 ± 12 mg/dL) compared to placebo after 29 weeks (40). However, because metformin was FDA-approved before the requirement of large scale CVOT's, available studies are relatively small and limited in answering the question of whether or not metformin confers true CV benefit. See Table 1A for a summary of the cardiovascular risks and benefits of diabetes medications that produce weight loss.

**Table 1A:** Risks and benefits of obesity pharmacotherapy, diabetes medications.

	**Composite CV outcomes**	**CV death**	**Non-fatal MI**	**Non-fatal Stroke**	**SBP**	**DBP**	**HR**	**HDL**	**LDL**	**TG**	**HF**
**Diabetes Medications**											
Liraglutide 1.8 mg	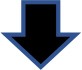	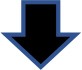	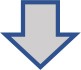	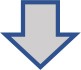	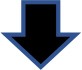	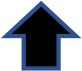	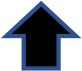	—	—	—	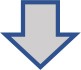
Liraglutide 3.0 mg	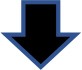	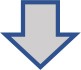	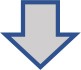	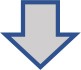	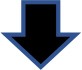	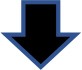	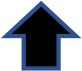	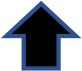	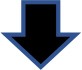	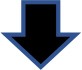	NR
Semaglutide	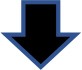	—	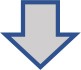	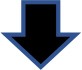	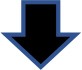	—	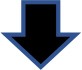	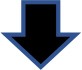	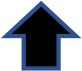	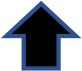	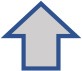
Exenatide	—	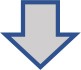	—	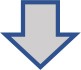	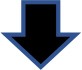	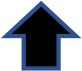	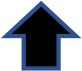	NR	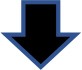	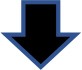	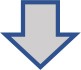
Dulaglutide	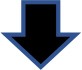	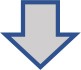	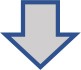	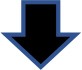	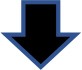	—	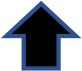	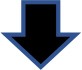	NR	NR	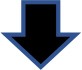
Lixisenatide	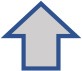	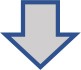	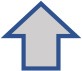	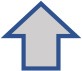	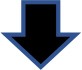	NR	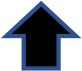	NR	NR	NR	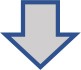
Metformin	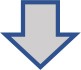	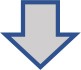	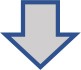	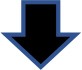	NR	NR	NR	NR	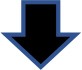	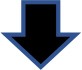	NR
SGLT2 Inhibitors	—	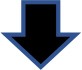	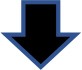	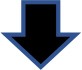	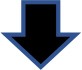	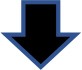	—	—	—	—	—

*Key: Black arrows 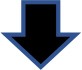

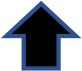
 indicate statistically significant change; gray arrows 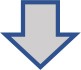

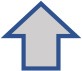
 indicate a non-statistically significant change; NR means not reported; — indicates reported; no change. Abbreviations: CV, cardiovascular; MI, myocardial infarction; SBP, systolic blood pressure; DBP, diastolic blood pressure; HR, heart rate; HDL, high density lipoprotein; LDL, low density lipoprotein; HF, heart failure; TG, triglycerides*.

## Stimulants

The second largest class of medications used for weight management are stimulants, most of which work to decrease appetite by increasing the release or maintenance of the catecholamines dopamine and norepinephrine.

### Phentermine

Phentermine, a norepinephrine reuptake inhibitor, was originally approved in 1959 by the United States FDA for the treatment of obesity in adults. Phentermine fell out of favor in the 1990s because of its association with fenfluramine in the combination pill “Fen-Phen.” Fenfluramine was found to be associated with valvular heart disease and pulmonary hypertension. Fenfluramine, which works primarily through serotonin receptors and to a less extent norepinephrine, will be discussed further below. Despite phentermine's past association with fenfluramine, phentermine is now the most commonly prescribed medication for the treatment of obesity in adults (41–43). Even with the high frequency in which phentermine is prescribed, no randomized control trials or prospective trials sensitive enough to determine the CV risks and benefits of this medication have been conducted. Phentermine has been associated with a reduction in LDL and increase in HDL cholesterol in a small randomized trial (44).

While phentermine is in the class of amphetamines, no adverse changes in either systolic or diastolic BP have been observed in studies of adults, including adults with pre-existing hypertension (44–47), nor in adolescents (48). Most studies show a decrease in blood pressure with phentermine, including in patients with pre-existing hypertension, and patients on high doses of phentermine (including 37.5 mg BID) (45, 47). It is unknown whether the decreased blood pressure would occur in the absence of weight loss, but decreased blood pressure with phentermine has been observed at 1 week, and with as little as 4% weight loss at 1 year (45, 47). Decreased blood pressure has remained in phentermine-treated patients at 2 years follow-up despite some weight regain (45). Additionally, in the 1–2 weeks after starting phentermine, when weight loss was small, blood pressure went down rather than up despite starting on maximum dose (45). In a cohort of 300 patients who achieved weight loss through a weight management clinic, the decrease in SBP and DBP was the same for phentermine-exposed and phentermine-unexposed patients achieving similar weight loss (45). Increased heart rate has not been observed in adult patients treated with phentermine alone (45). A transient elevation in HR has been observed in adolescents and warrants close monitoring by physicians (48). It is reasonable and preferred to check a patient's blood pressure soon after starting phentermine, because in theory adrenergic agents can increase blood pressure. Repeated observational studies do not show that phentermine increases blood pressure, but large prospective randomized studies with phentermine are needed.

Phentermine is approved for long-term use (>12 weeks) for weight management in combination with topiramate (Qsymia). Phentermine alone continues to be approved for short-term use (e.g., 12 weeks). In reality, phentermine is prescribed off-label to patients for longer than 12 weeks, and a recent study found improved weight loss and no adverse effects with this longer use (49). The safety profile of phentermine-topiramate is similar to that of phentermine, and has been associated with a decrease in BP compared to placebo (47, 50). Heart rate has been shown to increase in patients with phentermine/topiramate at 1 year follow-up by 0.6–1.6 bpm over placebo in patients with a resting heart rate <60 bpm; show no change in HR in patients with a resting heart rate between 60 and 90 bpm, and to decrease by 5–15 bpm in patients who had a resting heart rate >90 bpm at baseline (47). Topiramate will be reviewed separately below.

Diethylpropion is another stimulant medication that is FDA-approved for short-term use (<12 weeks) (51). Diethylpropion was first approved for use in humans in 1959 and, like phentermine, works as a norepinephrine-releasing agent. It has been associated with decreases in systolic BP and HR, and small increases in diastolic BP (52, 53). It has not been associated with valvular heart disease. This agent is not commonly used because of its inconvenient dosing (three to four times per day for the immediate-release formulation) and cost.

Stimulant medications that were developed to treat attention deficit and hyperactivity disorder (ADHD) are also used in weight management settings, particularly for the treatment of obesity in children and for the treatment of binge-eating disorder (54). Lisdexamfetamine (Vyvanse), FDA-approved for binge eating disorder in adults, has been associated with small increases in systolic BP (2 mmHg) and HR (6 bpm), and improvements in cholesterol (55–57). A review of over 1 million patient records of children and young adults (age 2–24 years) on stimulant medications for ADHD found no adverse CV events associated with use of these agents (58).

Overall, stimulants as a class of medications have not proven to be unsafe over time. While adrenergic agents may theoretically increase BP, data shows that systolic BP is consistently decreased with use of phentermine and diethylpropion. Years of ADHD medication use in children has shown no adverse CV events with these stimulant medications.

## Serotonin Receptor Modulators

Fenfluramine (Pondimin) is a sympathomimetic agent originally approved in 1959 as an appetite suppressant, and was used as a monotherapy and in combination with phentermine (Fen-Phen). Fenfluramine was found to be associated with valvular heart disease through its metabolite norfenfluramine, which caused activation of serotonin (5-HT2B) receptors present on cardiac cells (51). Fenfluramine caused appetite suppression primarily by increasing the release of serotonin in the brain, and to a lesser extent through norepinephrine release. Due to the associated cardiac valvular disease and pulmonary hypertension, fenfluramine, dexfenfluramine (its d-enantiomer), and fen-phen were removed from the market in 1997.

A newer agent, lorcaserin (Belviq), FDA-approved in 2012 for chronic use for obesity, suppresses appetite through modulation of serotonin receptors. Lorcaserin increases serotonin levels through activation of 5-HT2C serotonin receptors, rather than 5-HT2B serotonin receptors, which were activated by fenfluramine. The 5-HT2C receptors are found almost exclusively in the brain, specifically in the choroid plexus, hippocampus, cerebellum, amygdala, thalamus, and hypothalamus. Lorcaserin theoretically could bind to 5-HT2B receptors, however its affinity is over 100 times greater for 5-HT2C receptors than for 5-HT2B, and it has not been associated with valvular heart disease (59–61). A large, randomized double-blinded placebo-controlled study clinical trial was conducted to assess the effectiveness and safety of lorcaserin in high risk patients (patients with overweight or obesity and atherosclerotic disease or multiple CV risk factors (CAMELLIA-TIMI 61), Cardiovascular and Metabolic Effects of Lorcaserin in Overweight and Obesity Patients—Thrombolysis in Myocardial Infarction 61] (62). During 3 years of follow-up, lorcaserin was associated with slightly lower hazard ratio of cardiovascular outcomes, and a non-significant improvement in non-fatal MI and stroke, and non-significant increase in cardiovascular death (events infrequent in both groups) (62). Lorcaserin was associated with improved lipid profiles, heart rate, and improved blood pressure in both the CAMELLIA trial and previous trials (60–62).

Sibutramine (Meridia) is another appetite suppressant that works by increasing serotonin levels through decreasing reuptake of serotonin. Sibutramine was widely used until 2010, but was withdrawn from the market due to its association with CV events (MI, angina, arrhythmia) and stroke (63, 64). Unlike most other appetite suppressing medications, sibutramine has been associated with statistically significant increases in both HR (4 bpm), and systolic BP (1.7 mmHg) (51, 64). A small study did show that this increase in BP and HR was attenuated when patients adhered to supervised exercise therapy concomitant with sibutramine (65). Another centrally-acting anorectic medication was rimonabant (Acomplia), which was used in Europe in the early 2000's. While rimonabant was not associated with adverse CV events while it was on the market, it was withdrawn in Europe in 2009 because of adverse psychological effects (66).

The weight loss medications that have caused the most concern for CV safety have been from the class of medications that modulate the serotonin receptor. Lorcaserin is currently approved for use for weight loss and has not been associated with increased CV risk.

## Anti-Epileptic Medications

Topiramate was originally developed to treat seizures, and is most often used prophylactically for chronic migraine headache prevention. In 2012, the FDA approved the combination of phentermine-topiramate (Qsymia) for weight loss. While its mechanism of action for weight loss is not entirely clear, topiramate most likely causes weight loss by appetite suppression through modulation of gamma-aminobutyric acid (GABA) receptors, causing glutamate inhibition, as well as increased dopamine release (50, 67). Topiramate has also been found to have effects on neuropeptide-Y, a hormone that increases food consumption (67). Topiramate is used off-label as a single agent for weight loss and for the treatment of binge eating disorder (68). Topiramate has not been associated with increased CV risk, and has been associated with decreased systolic and diastolic BP (69).

Zonisamide is another anti-epileptic medication that has been used for weight loss, and has a similar purported mechanism of weight loss action to topiramate. Zonisamide is not approved for use for weight loss by the FDA. Zonisamide has not been found to have any adverse CV side effects, and has been associated with a decrease in BP and HR (43, 70). From a CV standpoint, topiramate and zonisamide appear to be safe, and do not appear to be associated with increased CV risk.

## Malabsorptive Agents and Fiber

Orlistat is the only medication approved for weight loss in persons younger than age 17 (71). Orlistat works by preventing absorption of about 30% of dietary fat consumed through pancreatic lipase inhibition. Because of this mechanism, common side effects include flatulence and fecal incontinence, especially in the setting of high amounts of dietary fat consumption. Orlistat has been associated with improved lipid profiles (beyond what can be explained by weight loss) and improved systolic and diastolic BP, even in patients with high CV risk (46, 72, 73). Orlistat can be purchased over the counter at a dose of 60 mg three times daily (Alli). The prescription dose of Orlistat is 120 mg three times daily.

Fiber supplements have been assessed for the efficacy for weight management efficacy, as high-fiber dietary patterns are generally associated with improved weight management. Psyllium fiber supplements in particular have been shown to have beneficial weight loss effects, thought to be due to improvements in satiety for several hours after consuming them (74). Psyllium and guar gum fiber supplements have also been shown to improve LDL levels and triglycerides (psyllium only), and increasing fiber in the diet is important for improving cholesterol (74, 75). Further, psyllium supplementation has been associated with improved blood pressure and resolution of metabolic syndrome (75). Orlistat and fiber appear to convey CV benefit, and also appear to have no known CV risk.

## Naltrexone/Bupropion (Contrave)

Naltrexone is an opioid receptor antagonist approved for opioid and alcohol dependence, and bupropion is a non-selective inhibitor of the dopamine and norepinephrine transporters approved for major depressive disorder, seasonal affective disorder, and smoking cessation (76). Wellbutrin has shown some efficacy in weight loss as a monotherapy, but naltrexone as monotherapy has not (77–79). In 2014, the combination of naltrexone extended-release (ER)/bupropion extended-release (ER) was FDA-approved for weight loss in adults (80). Weight loss from the combination of Naltrexone ER/bupropion ER appears to be greater than weight loss from each agent used as monotherapy (81).

In the CONTRAVE Obesity Research (COR) trials of adults with obesity [COR I, COR II, COR-Diabetes Mellitus (DM), and COR-Behavioral Modification (BMOD)], naltrexone-bupropion had slightly beneficial impacts on cholesterol levels, with placebo-subtracted increases in HDL of 2–5 mg/dl, changes in LDL ranging from a 4 mg/dl decrease to a 3 mg/dl increase (insignificant), and decreases in triglycerides of 8–10% after 1 year on the maximum approved dose (naltrexone 32 mg/bupropion 360 mg) (82–85). Overall, naltrexone/bupropion appears to have somewhat unfavorable impacts on BP and HR (86). In the COR trials, compared to placebo, those on naltrexone/bupropion had a 1.1–2.6 mmHg higher systolic blood pressure and 0.9–1.2 higher pulse after 1 year (82, 83, 85). These changes are similar to those seen in studies evaluating bupropion monotherapy for weight loss (78, 87), and could be due to bupropion's sympathomimetic effects (86). A meta-analysis examining serious adverse events from naltrexone was unable to analyze its effects on CVD development due to low numbers of events recorded (88). A large-scale RCT investigating the cardiovascular safety of bupropion for smoking cessation found no increased incidence of CVD (89). Finally, an FDA-mandated randomized non-inferiority cardiovascular outcomes trial in patients with pre-existing cardiovascular risk factors did not show excess risk for adverse CV events (90). However, the study was terminated at the 50% interim analysis due to inclusion of the 25% interim analyses on the patent publication, resulting in the potential for unblinding (76, 90). Therefore, non-inferiority could not be definitively determined. Overall, naltrexone-bupropion does appear to consistently cause a small increase in systolic BP, likely driven by bupropion, but in several recent studies, naltrexone-bupropion did not increase adverse cardiovascular outcomes.

## Leptin

Leptin, the neuro-hormone produced by adipocytes, primarily acts on the hypothalamus to regulate appetite and energy expenditure. In weight-reduced adults, decreased leptin signaling blunts feeding inhibition (91). Although recombinant leptin therapy has not effectively resulted in weight loss for patients with exogenous obesity, it has been effective in patients with congenital leptin deficiency (92–96). First described in humans in 1997, congenital leptin deficiency is associated with early-onset severe obesity, hyperphagia, lower energy expenditure, impaired satiety, and low leptin levels (97). In patients with congenital leptin deficiency, treatment with daily subcutaneous recombinant leptin therapy has been found to decrease overall body weight, body fat percentage, appetite, and overall energy intake (95, 96). Leptin replacement has also increased insulin sensitivity, decreased hepatic insulin removal, and improved glucose control in patients with leptin deficiency (96). In addition, leptin replacement has been found to decrease triglycerides and increase HDL, reverse hypogonadotropic hypogonadism, increase growth, and regulate thyroid stimulating hormone (96). While leptin therapy is not currently approved in the absence of congenital leptin deficiency, leptin therapy appears to reduce CV risk factors in those with leptin deficiency. See Table 1B for a summary of CV effects of non-diabetes medications that cause weight loss.

**Table 1B:** Risks and benefits of pharmacotherapy used for weight loss.

	**Composite CV outcomes**	**CV death**	**Non-fatal MI**	**Non-fatal Stroke**	**SBP**	**DBP**	**HR**	**HDL**	**LDL**	**TG**	**HF**
**STIMULANT MEDICATIONS**		
Phentermine/Topiramate	—	—	—	—	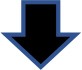	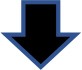	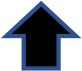	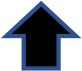	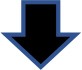	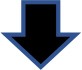	—
Phentermine	NR	NR	NR	NR	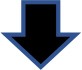	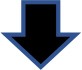	—	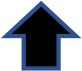	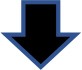	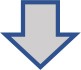	NR
Diethylpropion	NR	NR	NR	NR	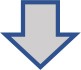	—	—	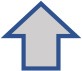	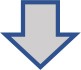	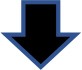	NR
Lisdexamfetamine	—	—	—	—	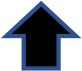	—	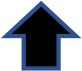	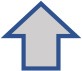	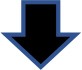	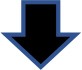	—
Lorcaserin	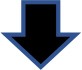	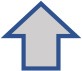	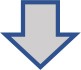	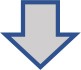	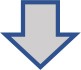	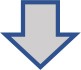	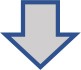	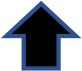	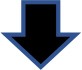	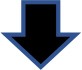	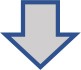
Topiramate	—	—	—	—	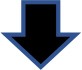	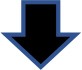	—	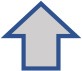	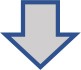	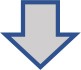	—
Zonisamide	NR	NR	NR	NR	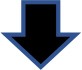	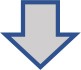	—	—	—	—	NR
Orlistat	NR	NR	NR	NR	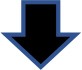	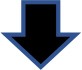	NR	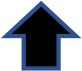	—	—	NR
Naltrexone/Bupropion^*^	—	—	—	—	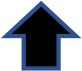	—	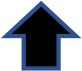	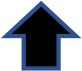	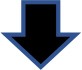	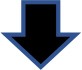	—
Bupropion	NR	NR	NR	NR	—	—	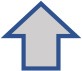	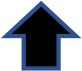	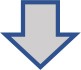	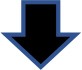	NR

*Key: Black arrows 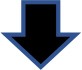

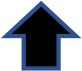
 indicate statistically significant change; gray arrows 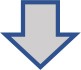

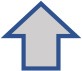
 indicate a non-statistically significant change; NR means not reported; — indicates reported; no change*.

* *Study reporting major CV outcomes discontinued due to potential for non-blinding. Abbreviations: CV, cardiovascular; MI, myocardial infarction; SBP, systolic blood pressure; DBP, diastolic blood pressure; HR, heart rate; HDL, high density lipoprotein; LDL, low density lipoprotein; HF, heart failure; TG, triglycerides*.

## Conclusion

Given the high prevalence of obesity and the many negative health consequences of obesity, effective and safe pharmacotherapy is urgently needed in both children and adults. Consequently, obesity places persons at higher risk for CV disease, thus it is importance to understand the CV health implications of weight loss medications. Most anti-obesity medications appear to have safe, if not beneficial, CV risk profiles. GLP-1 agonists consistently show improved markers of CV health, as well as decreased rates of CV events. Further, SGLT2 inhibitors have shown improved markers of CV health, improved cardiovascular CV outcomes, and decreased adverse CV events. While DPP-4 inhibitors appear to worsen rates of hospitalization in those with heart failure, they had no additional adverse effects on other CV outcomes. Simulant medications, antiepileptic medications, and orlistat have favorable effects on markers of CV health (namely improved BP and cholesterol) and no adverse cardiovascular events. Naltrexone/bupropion improves cholesterol, increases BP slightly, but has not been shown to improve or worsen risk for CV events. The medications known to cause severe CV consequences (fenfluramine, dexfenfluramine, and sibutramine) primarily involve serotonin receptor modulation and have been removed from the market. These adverse effects have not been seen with lorcaserin likely due to its mechanism working through a different serotonin receptor. Unfortunately, placebo-controlled studies with long-term follow-up do not exist for all weight loss medications and do not exist for all populations such as children and adolescents, persons with overweight, and persons with existing CV disease. Future studies are needed to address these gaps as the demand for obesity pharmacotherapy is likely to become even greater. As with all prescribing, clinicians should understand their patients' individual risk profiles and always discuss risks and benefits with patients.

## Author Contributions

SR wrote the section on GLP1 agonists, DDP-4 inhibitors, and metformin. EB wrote the section on naltrexone-bupropion. MO wrote the section on leptin. JR wrote the section on SGLT-2 inhibitors and phentermine. CB wrote all remaining sections, reviewed and edited sections written by others, and created the tables. All authors reviewed and edited the final product.

### Conflict of Interest

JR receives support from Boehringer Ingelheim Pharmaceuticals in the form of drug/placebo. MO receives research support from Vivus, Inc. The funders had no role in the study design, data collection and analysis, decision to publish, or preparation of the manuscript. EB is an investigator for a study funded by Novo Nordisk. He does not have access to the study data. The remaining authors declare that the research was conducted in the absence of any commercial or financial relationships that could be construed as a potential conflict of interest.

## References

[1] The Lancet Public Health Tackling obesity seriously: the time has come. Lancet Public Health. (2018) 3:e153 10.1016/S2468-2667(18)30053-7 29627076

[2] Afshin A Forouzanfar MH Reitsma MB Sur P Estep K Lee A. Health effects of overweight and obesity in 195 countries over 25 years. N Engl J Med. (2017) 377:13–27. 10.1056/NEJMoa1614362 28604169 PMC5477817

[3] Connolly HM Crary JL McGoon MD Hensrud DD Edwards BS Edwards WD. Valvular heart disease associated with fenfluramine-phentermine. N Engl J Med. (1997) 337:581–8. 10.1056/NEJM199708283370901 9271479

[4] Apovian CM Aronne LJ Bessesen DH McDonnell ME Murad MH Pagotto U. Pharmacological management of obesity: an endocrine society clinical practice guideline. J Clin Endocrinol Metab. (2015) 100:342–62. 10.1210/jc.2014-3415 25590212

[5] Jensen MD Ryan DH Apovian CM Ard JD Comuzzie AG Donato KA. 2013 AHA/ACC/TOS guideline for the management of overweight and obesity in adults: a report of the American College of Cardiology/American Heart Association Task Force on Practice Guidelines and The Obesity Society. J Am Coll Cardiol. (2014) 63(25 Pt B):2985–3023. 10.1161/01.cir.0000437739.71477.ee 24239920

[6] FDA Diabetes Mellitus — Evaluating Cardiovascular Risk in New Antidiabetic Therapies to Treat Type 2 Diabetes. Center for Drug Evaluation and Research (2008).

[7] Roder ME. Major adverse cardiovascular event reduction with GLP-1 and SGLT2 agents: evidence and clinical potential. Ther Adv Chronic Dis. (2018) 9:33–50. 10.1177/2040622317735283 29344329 PMC5761941

[8] Farr OM Sofopoulos M Tsoukas MA Dincer F Thakkar B Sahin-Efe A. GLP-1 receptors exist in the parietal cortex, hypothalamus and medulla of human brains and the GLP-1 analogue liraglutide alters brain activity related to highly desirable food cues in individuals with diabetes: a crossover, randomised, placebo-controlled trial. Diabetologia. (2016) 59:954–65. 10.1007/s00125-016-3874-y 26831302 PMC4826792

[9] Pi-Sunyer X Astrup A Fujioka K Greenway F Halpern A Krempf M. A randomized, controlled trial of 3.0 mg of liraglutide in weight management. N Engl J Med. (2015) 373:11–22. 10.1056/NEJMoa1411892 26132939

[10] Davies MJ Aronne LJ Caterson ID Thomsen AB Jacobsen PB Marso SP. Liraglutide and cardiovascular outcomes in adults with overweight or obesity: A post hoc analysis from SCALE randomized controlled trials. Diabetes Obes Metab. (2018) 20:734–9. 10.1111/dom.13125 28950422 PMC5836948

[11] Marso SP Daniels GH Brown-Frandsen K Kristensen P Mann JFE Nauck MA. Liraglutide and cardiovascular outcomes in type 2 diabetes. N Engl J Med. (2016) 375:311–22. 10.1056/NEJMoa1603827 27295427 PMC4985288

[12] Marso SP Bain SC Consoli A Eliaschewitz FG Jodar E Leiter LA. Semaglutide and cardiovascular outcomes in patients with type 2 diabetes. N Engl J Med. (2016) 375:1834–44. 10.1056/NEJMoa1607141 27633186

[13] Gerstein HC Colhoun HM Dagenais GR Diaz R Lakshmanan M Pais P. Dulaglutide and cardiovascular outcomes in type 2 diabetes (REWIND): a double-blind, randomised placebo-controlled trial. Lancet. (2019) 394:121–30. 10.1016/S0140-6736(19)31149-3 31189511

[14] Holman RR Bethel MA Mentz RJ Thompson VP Lokhnygina Y Buse JB. Effects of once-weekly exenatide on cardiovascular outcomes in type 2 diabetes. N Engl J Med. (2017) 377:1228–39. 10.1056/NEJMoa1612917 28910237 PMC9792409

[15] Pfeffer MA Claggett B Diaz R Dickstein K Gerstein HC Kober LV. Lixisenatide in patients with type 2 diabetes and acute coronary syndrome. N Engl J Med. (2015) 373:2247–57. 10.1056/NEJMoa1509255 26630143

[16] Sun F Wu S Wang J Guo S Chai S Yang Z. Effect of glucagon-like peptide-1 receptor agonists on lipid profiles among type 2 diabetes: a systematic review and network meta-analysis. Clin Ther. (2015) 37:225–41.e8. 10.1016/j.clinthera.2014.11.008 25554560

[17] Kumarathurai P Anholm C Larsen BS Olsen RH Madsbad S Kristiansen O. Effects of liraglutide on heart rate and heart rate variability: a randomized, double-blind, placebo-controlled crossover study. Diabetes Care. (2017) 40:117–24. 10.2337/dc16-1580 27797930

[18] Zhao X Huang K Zheng M Duan J. Effect of liraglutide on blood pressure: a meta-analysis of liraglutide randomized controlled trials. BMC Endocr Disord. (2019) 19:4. 10.1186/s12902-018-0332-5 30616638 PMC6323665

[19] Margulies KB Hernandez AF Redfield MM Givertz MM Oliveira GH Cole R. Effects of liraglutide on clinical stability among patients with advanced heart failure and reduced ejection fraction: a randomized clinical trial. JAMA. (2016) 316:500–8. 10.1001/jama.2016.10260 27483064 PMC5021525

[20] Plutzky J Garber A Falahati A Toft AD Poulter NR. Reductions in lipids and CV risk markers in patients with type 2 diabetes treated with liraglutide: a meta-analysis. Can J Diabetes. (2009) 33:209–10. 10.1016/S1499-2671(09)33072-5 25998594

[21] Sun F Wu S Guo S Yu K Yang Z Li L. Impact of GLP-1 receptor agonists on blood pressure, heart rate and hypertension among patients with type 2 diabetes: a systematic review and network meta-analysis. Diabetes Res Clin Pract. (2015) 110:26–37. 10.1016/j.diabres.2015.07.015 26358202

[22] Matikainen N Soderlund S Bjornson E Pietilainen K Hakkarainen A Lundbom N. Liraglutide treatment improves postprandial lipid metabolism and cardiometabolic risk factors in humans with adequately controlled type 2 diabetes: a single-centre randomized controlled study. Diabetes Obes Metab. (2019) 21:84–94. 10.1111/dom.13487 30073766 PMC6585708

[23] Rondinelli M Rossi A Gandolfi A Saponaro F Bucciarelli L Adda G. Use of liraglutide in the real world and impact at 36 months on metabolic control, weight, lipid profile, blood pressure, heart rate, and renal function. Clin Ther. (2017) 39:159–69. 10.1016/j.clinthera.2016.11.001 27939305

[24] Merovci A Solis-Herrera C Daniele G Eldor R Fiorentino TV Tripathy D. Dapagliflozin improves muscle insulin sensitivity but enhances endogenous glucose production. J Clin Investig. (2014) 124:509–14. 10.1172/JCI70704 24463448 PMC3904617

[25] List JF Woo V Morales E Tang W Fiedorek FT. Sodium-glucose cotransport inhibition with dapagliflozin in type 2 diabetes. Diabetes Care. (2009) 32:650–7. 10.2337/dc08-1863 19114612 PMC2660449

[26] Chao EC Henry RR. SGLT2 inhibition — a novel strategy for diabetes treatment. Nat Rev Drug Discov. (2010) 9:551–9. 10.1038/nrd3180 20508640

[27] Nauck MA Del Prato S Meier JJ Duran-Garcia S Rohwedder K Elze M. Dapagliflozin versus glipizide as add-on therapy in patients with type 2 diabetes who have inadequate glycemic control with metformin: a randomized, 52-week, double-blind, active-controlled noninferiority trial. Diabetes Care. (2011) 34:2015–22. 10.2337/dc11-0606 21816980 PMC3161265

[28] Kadowaki T Haneda M Inagaki N Terauchi Y Taniguchi A Koiwai K. Efficacy and safety of empagliflozin monotherapy for 52 weeks in japanese patients with type 2 diabetes: a randomized, double-blind, parallel-group study. Adv Ther. (2015) 32:306–18. 10.1007/s12325-015-0198-0 25845768

[29] Ross S Thamer C Cescutti J Meinicke T Woerle HJ Broedl UC Efficacy and safety of empagliflozin twice daily compared with once daily in patients with type 2 diabetes inadequately controlled on metformin: a 16-week, randomized, placebo-controlled trial. Diabetes Obes Metab. (2015) 17:699–702. 10.1111/dom.12469 25827441

[30] Chilton R Tikkanen I Cannon CP Crowe S Woerle HJ Broedl UC. Effects of empagliflozin on blood pressure and markers of arterial stiffness and vascular resistance in patients with type 2 diabetes. Diabetes Obes Metab. (2015) 17:1180–93. 10.1111/dom.12572 26343814 PMC5057299

[31] Zinman B Wanner C Lachin JM Fitchett D Bluhmki E Hantel S. Empagliflozin, cardiovascular outcomes, and mortality in type 2 diabetes. N Engl J Med. (2015) 373:2117–28. 10.1056/NEJMoa1504720 26378978

[32] DeFronzo RA Hompesch M Kasichayanula S Liu X Hong Y Pfister M. Characterization of renal glucose reabsorption in response to dapagliflozin in healthy subjects and subjects with type 2 diabetes. Diabetes Care. (2013) 36:3169–76. 10.2337/dc13-0387 23735727 PMC3781504

[33] Ferrannini E Baldi S Frascerra S Astiarraga B Barsotti E Clerico A. Renal handling of ketones in response to sodium-glucose cotransporter 2 inhibition in patients with type 2 diabetes. Diabetes Care. (2017) 40:771–6. 10.2337/dc16-2724 28325783

[34] Cherney DZ Perkins BA Soleymanlou N Har R Fagan N Johansen OE. The effect of empagliflozin on arterial stiffness and heart rate variability in subjects with uncomplicated type 1 diabetes mellitus. Cardiovasc Diabetol. (2014) 13:28. 10.1186/1475-2840-13-28 24475922 PMC3915232

[35] Abdul-Ghani MA Norton L DeFronzo RA. Renal sodium-glucose cotransporter inhibition in the management of type 2 diabetes mellitus. Am J Physiol Renal Physiol. (2015) 309:F889–900. 10.1152/ajprenal.00267.2015 26354881 PMC4669360

[36] Rena G Hardie DG Pearson ER. The mechanisms of action of metformin. Diabetologia. (2017) 60:1577–85. 10.1007/s00125-017-4342-z 28776086 PMC5552828

[37] Effect of intensive blood-glucose control with metformin on complications in overweight patients with type 2 diabetes (UKPDS 34) UK Prospective Diabetes Study (UKPDS) group. Lancet. (1998) 352:854–65. 10.1016/S0140-6736(98)07037-8 9742977

[38] Boussageon R Supper I Bejan-Angoulvant T Kellou N Cucherat M Boissel JP. Reappraisal of metformin efficacy in the treatment of type 2 diabetes: a meta-analysis of randomised controlled trials. PLoS Med. (2012) 9:e1001204. 10.1371/journal.pmed.1001204 22509138 PMC3323508

[39] Griffin SJ Leaver JK Irving GJ. Impact of metformin on cardiovascular disease: a meta-analysis of randomised trials among people with type 2 diabetes. Diabetologia. (2017) 60:1620–9. 10.1007/s00125-017-4337-9 28770324 PMC5552849

[40] DeFronzo RA Goodman AM. Efficacy of metformin in patients with non-insulin-dependent diabetes mellitus. The multicenter metformin study group. N Engl J Med. (1995) 333:541–9. 10.1056/NEJM199508313330902 7623902

[41] Hendricks EJ Rothman RB Greenway FL. How physician obesity specialists use drugs to treat obesity. Obesity. (2009) 17:1730–5. 10.1038/oby.2009.69 19300434

[42] Stafford RS Radley DC. National trends in antiobesity medication use. Archiv Inter Med. (2003) 163:1046–50. 10.1001/archinte.163.9.1046 12742801

[43] Domecq JP Prutsky G Leppin A Sonbol MB Altayar O Undavalli C. Clinical review: Drugs commonly associated with weight change: a systematic review and meta-analysis. J Clin Endocrinol Metab. (2015) 100:363–70. 10.1210/jc.2014-3421 25590213 PMC5393509

[44] Kang JG Park CY Kang JH Park YW Park SW. Randomized controlled trial to investigate the effects of a newly developed formulation of phentermine diffuse-controlled release for obesity. Diabetes Obes Metab. (2010) 12:876–2. 10.1111/j.1463-1326.2010.01242.x 20920040

[45] Hendricks EJ Greenway FL Westman EC Gupta AK. Blood pressure and heart rate effects, weight loss and maintenance during long-term phentermine pharmacotherapy for obesity. Obesity. (2011) 19:2351–60. 10.1038/oby.2011.94 21527891

[46] Siebenhofer A Jeitler K Horvath K Berghold A Posch N Meschik J. Long-term effects of weight-reducing drugs in people with hypertension. Cochrane Database Syst Rev. (2016) 3:Cd007654. 10.1002/14651858.CD007654.pub4 26934640

[47] Jordan J Astrup A Engeli S Narkiewicz K Day WW Finer N. Cardiovascular effects of phentermine and topiramate: a new drug combination for the treatment of obesity. J Hypertens. (2014) 32:1178–88. 10.1097/HJH.0000000000000145 24621808 PMC4011567

[48] Ryder JR Kaizer A Rudser KD Gross A Kelly AS Fox CK. Effect of phentermine on weight reduction in a pediatric weight management clinic. Int J Obes. (2017) 41:90–3. 10.1038/ijo.2016.185 27773937 PMC5891125

[49] Lewis KH Fischer H Ard J Barton L Bessesen DH Daley MF. Safety and effectiveness of longer-term phentermine use: clinical outcomes from an electronic health record cohort. Obesity. (2019) 27:591–602. 10.1002/oby.22430 30900410

[50] Ellinger LK Ipema HJ Stachnik JM. Efficacy of metformin and topiramate in prevention and treatment of second-generation antipsychotic-induced weight gain. Ann Pharmacother. (2010) 44:668–79. 10.1345/aph.1M550 20233913

[51] Farah D Fonseca MCM. Short-term evidence in adults of anorexigenic drugs acting in the central nervous system: a meta-analysis. Clin Ther. (2019) 41:1798–815. 10.1016/j.clinthera.2019.06.005 31351676

[52] Suplicy H Boguszewski CL dos Santos CM do Desterro de Figueiredo M Cunha DR Radominski R. A comparative study of five centrally acting drugs on the pharmacological treatment of obesity. Int J Obesity. (2014) 38:1097–103. 10.1038/ijo.2013.225 24287940

[53] Cercato C Roizenblatt VA Leanca CC Segal A Loppes Filho AP Mancini MC. A randomized double-blind placebo-controlled study of the long-term efficacy and safety of diethylpropion in the treatment of obese subjects. Int J Obes. (2009) 33:857–65. 10.1038/ijo.2009.124 19564877

[54] McElroy SL Hudson JI Gasior M Herman BK Radewonuk J Wilfley D. Time course of the effects of lisdexamfetamine dimesylate in two phase 3, randomized, double-blind, placebo-controlled trials in adults with binge-eating disorder. Int J Eat Dis. (2017) 50:884–92. 10.1002/eat.22722 28481434 PMC5573905

[55] Hudson JI McElroy SL Ferreira-Cornwell MC Radewonuk J Gasior M. Efficacy of lisdexamfetamine in adults with moderate to severe binge-eating disorder: a randomized clinical trial. JAMA Psychiatry. (2017) 74:903–10. 10.1001/jamapsychiatry.2017.1889 28700805 PMC5710231

[56] Charach G Kaysar N Grosskopf I Rabinovich A Weintraub M. Methylphenidate has positive hypocholesterolemic and hypotriglyceridemic effects: new data. J Clin Pharmacol. (2009) 49:848–51. 10.1177/0091270009336736 19553406

[57] Coghill DR Caballero B Sorooshian S Civil R. A systematic review of the safety of lisdexamfetamine dimesylate. CNS Drugs. (2014) 28:497–511. 10.1007/s40263-014-0166-2 24788672 PMC4057639

[58] FDA FDA Drug Safety Communication: Safety Review Update of Medications Used to treat Attention-Deficit/Hyperactivity Disorder (ADHD) in Children and Young Adults. FDA (2018).

[59] Farr OM Upadhyay J Gavrieli A Camp M Spyrou N Kaye H. Lorcaserin administration decreases activation of brain centers in response to food cues and these emotion- and salience-related changes correlate with weight loss effects: a 4-week-long randomized, placebo-controlled, double-blind clinical trial. Diabetes. (2016) 65:2943–53. 10.2337/db16-0635 27385157 PMC5033259

[60] Smith SR Weissman NJ Anderson CM Sanchez M Chuang E Stubbe S. Multicenter, placebo-controlled trial of lorcaserin for weight management. N Engl J Med. (2010) 363:245–56. 10.1056/NEJMoa0909809 20647200

[61] Aronne L Shanahan W Fain R Glicklich A Soliman W Li Y. Safety and efficacy of lorcaserin: a combined analysis of the BLOOM and BLOSSOM trials. Postgr Med. (2014) 126:7–18. 10.3810/pgm.2014.10.2817 25414931

[62] Bohula EA Wiviott SD McGuire DK Inzucchi SE Kuder J Im K Cardiovascular safety of lorcaserin in overweight or obese patients. N Engl J Med. (2018) 379:1107–17. 10.1056/NEJMoa1808721 30145941

[63] FDA Meridia (Sibutramine Hydrochloride): Follow-Up to an Early Communication About an Ongoing Safety Review (2010).

[64] Scheen AJ. Sibutramine on cardiovascular outcome. Diabetes Care. (2011) 34(Suppl. 2):S114–9. 10.2337/dc11-s205 21525441 PMC3632147

[65] Berube-Parent S Prud'homme D St-Pierre S Doucet E Tremblay A. Obesity treatment with a progressive clinical tri-therapy combining sibutramine and a supervised diet–exercise intervention. Int J obes Related Metab Dis. (2001) 25:1144–53. 10.1038/sj.ijo.0801677 11477499

[66] Christensen R Kristensen PK Bartels EM Bliddal H Astrup A. Efficacy and safety of the weight-loss drug rimonabant: a meta-analysis of randomised trials. Lancet. (2007) 370:1706–13. 10.1016/S0140-6736(07)61721-8 18022033

[67] Cosentino G Conrad AO Uwaifo GI. Phentermine and topiramate for the management of obesity: a review. Drug Des Dev Ther. (2013) 7:267–78. 10.2147/DDDT.S31443 23630412 PMC3623549

[68] Kalaria SN McElroy SL Gobburu J Gopalakrishnan M. An innovative disease-drug-trial framework to guide binge eating disorder drug development: a case study for topiramate. Clin Transl Sci. (2019). 10.1111/cts.12682. [Epub ahead of print]. 31386273 PMC6951469

[69] Moradi S Kerman SR Mollabashi M. The effect of topiramate on weight loss in patients with type 2 diabetes. J Res Med Sci. (2013) 18:297–302. 24124426 PMC3793374

[70] Gadde KM Kopping MF Wagner HR II Yonish GM Allison DB Bray GA. Zonisamide for weight reduction in obese adults: a 1-year randomized controlled trial. Archiv Int Med. (2012) 172:1557–64. 10.1001/2013.jamainternmed.99 23147455 PMC3753218

[71] Mead E Atkinson G Richter B Metzendorf MI Baur L Finer N. Drug interventions for the treatment of obesity in children and adolescents. Cochrane Database Syst Rev. (2016) 11:Cd012436. 10.1002/14651858.CD012436 27899001 PMC6472619

[72] LeBlanc ES O'Connor E Whitlock EP Patnode CD Kapka T. Effectiveness of primary care–relevant treatments for obesity in adults: a systematic evidence review for the U.S. preventive services task force. Annals Int Med. (2011) 155:434–47. 10.7326/0003-4819-155-7-201110040-00006 21969342

[73] Davidson MH Hauptman J DiGirolamo M Foreyt JP Halsted CH Heber D. Weight control and risk factor reduction in obese subjects treated for 2 years with orlistat: a randomized controlled trial. JAMA. (1999) 281:235–42. 10.1001/jama.281.3.235 9918478

[74] Lambeau KV McRorie JW Jr. Fiber supplements and clinically proven health benefits: How to recognize and recommend an effective fiber therapy. J Am Assoc Nurse Pract. (2017) 29:216–23. 10.1002/2327-6924.12447 28252255 PMC5413815

[75] McRorie JW Jr. Evidence-based approach to fiber supplements and clinically meaningful health benefits, part 2: what to look for and how to recommend an effective fiber therapy. Nutr Today. (2015) 50:90–7. 10.1097/NT.0000000000000089 25972619 PMC4415970

[76] Bello NT. Update on drug safety evaluation of naltrexone/bupropion for the treatment of obesity. Expert Opin Drug Saf. (2019) 18:549–52. 10.1080/14740338.2019.1618268 31092063

[77] Li Z Maglione M Tu W Mojica W Arterburn D Shugarman LR. Meta-analysis: pharmacologic treatment of obesity. Ann Int Med. (2005) 142:532–46. 10.7326/0003-4819-142-7-200504050-00012 15809465

[78] Anderson JW Greenway FL Fujioka K Gadde KM McKenney J O'Neil PM. Bupropion SR enhances weight loss: a 48-week double-blind, placebo- controlled trial. Obes Res. (2002) 10:633–41. 10.1038/oby.2002.86 12105285

[79] Malcolm R O'Neil PM Sexauer JD Riddle FE Currey HS Counts C. A controlled trial of naltrexone in obese humans. Int J Obes. (1985) 9:347–53. 3908352

[80] Yanovski SZ Yanovski JA. Naltrexone extended-release plus bupropion extended-release for treatment of obesity. JAMA. (2015) 313:1213–4. 10.1001/jama.2015.1617 25803343 PMC4993523

[81] Fujioka K Plodkowski R O'Neil PM Gilder K Walsh B Greenway FL. The relationship between early weight loss and weight loss at 1 year with naltrexone ER/bupropion ER combination therapy. Int J Obes. (2016) 40:1369–75. 10.1038/ijo.2016.67 27328752

[82] Apovian CM Aronne L Rubino D Still C Wyatt H Burns C. A randomized, phase 3 trial of naltrexone SR/bupropion SR on weight and obesity-related risk factors (COR-II). Obesity. (2013) 21:935–43. 10.1002/oby.20309 23408728 PMC3739931

[83] Greenway FL Fujioka K Plodkowski RA Mudaliar S Guttadauria M Erickson J. Effect of naltrexone plus bupropion on weight loss in overweight and obese adults (COR-I): a multicentre, randomised, double-blind, placebo-controlled, phase 3 trial. Lancet. (2010) 376:595–605. 10.1016/S0140-6736(10)60888-4 20673995

[84] Hollander P Gupta AK Plodkowski R Greenway F Bays H Burns C. Effects of naltrexone sustained-release/bupropion sustained-release combination therapy on body weight and glycemic parameters in overweight and obese patients with type 2 diabetes. Diabetes Care. (2013) 36:4022–9. 10.2337/dc13-0234 24144653 PMC3836105

[85] Wadden TA Foreyt JP Foster GD Hill JO Klein S O'Neil PM. Weight loss with naltrexone SR/bupropion SR combination therapy as an adjunct to behavior modification: the COR-BMOD trial. Obesity. (2011) 19:110–20. 10.1038/oby.2010.147 20559296 PMC4459776

[86] Vorsanger MH Subramanyam P Weintraub HS Lamm SH Underberg JA Gianos E. Cardiovascular effects of the new weight loss agents. J Am Coll Cardiol. (2016) 68:849–59. 10.1016/j.jacc.2016.06.007 27539178

[87] Thase ME Haight BR Johnson MC Hunt T Krishen A Fleck RJ. A randomized, double-blind, placebo-controlled study of the effect of sustained-release bupropion on blood pressure in individuals with mild untreated hypertension. J Clin Psychopharmacol. (2008) 28:302–7. 10.1097/JCP.0b013e318172424e 18480687

[88] Bolton M Hodkinson A Boda S Mould A Panagioti M Rhodes S. Serious adverse events reported in placebo randomised controlled trials of oral naltrexone: a systematic review and meta-analysis. BMC Med. (2019) 17:10. 10.1186/s12916-018-1242-0 30642329 PMC6332608

[89] Benowitz NL Pipe A West R Hays JT Tonstad S McRae T. Cardiovascular safety of varenicline, bupropion, and nicotine patch in smokers: a randomized clinical trial. JAMA Int Med. (2018) 178:622–31. 10.1001/jamainternmed.2018.0397 29630702 PMC6145797

[90] Nissen SE Wolski KE Prcela L Wadden T Buse JB Bakris G. Effect of naltrexone-bupropion on major adverse cardiovascular events in overweight and obese patients with cardiovascular risk factors: a randomized clinical trial. JAMA. (2016) 315:990–1004. 10.1001/jama.2016.1558 26954408

[91] Kissileff HR Thornton JC Torres MI Pavlovich K Mayer LS Kalari V. Leptin reverses declines in satiation in weight-reduced obese humans. Am J Clin Nutr. (2012) 95:309–17. 10.3945/ajcn.111.012385 22237063 PMC3260066

[92] Tam CS Lecoultre V Ravussin E. Novel strategy for the use of leptin for obesity therapy. Expert Opin Biol Ther. (2011) 11:1677–85. 10.1517/14712598.2011.619974 21910668 PMC4220672

[93] Shetty GK Matarese G Magkos F Moon HS Liu X Brennan AM Leptin administration to overweight and obese subjects for six months increases free leptin concentrations but does not alter circulating hormones of the thyroid and IGF axes during weight loss induced by a mild hypocaloric diet. Eur J Endocrinol. (2011) 165:249–54. 10.1530/EJE-11-0252 21602313 PMC3159386

[94] Mittendorfer B Horowitz JF DePaoli AM McCamish MA Patterson BW Klein S Recombinant human leptin treatment does not improve insulin action in obese subjects with type 2 diabetes. Diabetes. (2011) 60:1474–7. 10.2337/db10-1302 21411512 PMC3292320

[95] Farooqi IS Jebb SA Langmack G Lawrence E Cheetham CH Prentice AM. Effects of recombinant leptin therapy in a child with congenital leptin deficiency. N Engl J Med. (1999) 341:879–84. 10.1056/NEJM199909163411204 10486419

[96] Paz-Filho G Wong ML Licinio J. Ten years of leptin replacement therapy. Obes Rev. (2011) 12:e315–23. 10.1111/j.1467-789X.2010.00840.x 21410864

[97] Montague CT Farooqi IS Whitehead JP Soos MA Rau H Wareham NJ. Congenital leptin deficiency is associated with severe early-onset obesity in humans. Nature. (1997) 387:903–8. 10.1038/43185 9202122

